# Supplementary material for: Plant grafting relieves asymmetry of jasmonic acid response induced by wounding between scion and rootstock in tomato hypocotyl
Source: PLoS One. 2020 Nov 24;15(11):e0241317. doi: 10.1371/journal.pone.0241317 (PMC7685457; doi:10.1371/journal.pone.0241317)
Supplement: S1 Table — (PDF) [file pone.0241317.s001.pdf]

S1 Table

Components	Groups	Time (h)	Mean±SE
JA	Separated Top	0	3.65±0.06
	Separated Top	0.5	5.44±0.39
	Separated Top	1	31.10±1.59
	Separated Top	2	37.77±1.77
	Separated Top	6	2.54±0.15
	Separated Top	12	5.30±0.46
	Separated Bottom	0	3.74±0.07
	Separated Bottom	0.5	2.59±0.10
	Separated Bottom	1	15.00±0.70
	Separated Bottom	2	5.63±0.28
	Separated Bottom	6	2.53±0.21
	Separated Bottom	12	3.40±0.09
	Grafted Top (Scion)	0.5	2.65±0.46
	Grafted Top (Scion)	1	27.80±1.97
	Grafted Top (Scion)	2	10.48±0.19
	Grafted Top (Scion)	6	4.28±0.14
	Grafted Top (Scion)	12	4.22±0.04
	Grafted bottom（Rootstock）	0.5	3.98±0.22
	Grafted bottom（Rootstock）	1	12.17±0.24
	Grafted bottom（Rootstock）	2	9.74±0.30
	Grafted bottom（Rootstock）	6	5.51±0.28
	Grafted bottom（Rootstock）	12	4.06±0.08
JA-ILE	Separated Top	0	12.43±0.05
	Separated Top	0.5	3.66±0.21
	Separated Top	1	9.71±0.16
	Separated Top	2	7.22±0.13
	Separated Top	6	1.13±0.13
	Separated Top	12	1.19±0.04
	Separated Bottom	0	12.45±0.03
	Separated Bottom	0.5	1.82±0.39
	Separated Bottom	1	5.43±0.22
	Separated Bottom	2	2.37±0.43
	Separated Bottom	6	1.34±0.09
	Separated Bottom	12	0.68±0.04
	Grafted Top (Scion)	0.5	2.81±0.28
	Grafted Top (Scion)	1	6.24±0.01
	Grafted Top (Scion)	2	1.44±0.19
	Grafted Top (Scion)	6	12.86±0.04
	Grafted Top (Scion)	12	12.70±0.01
	Grafted bottom（Rootstock）	0.5	1.82±0.39
	Grafted bottom（Rootstock）	1	5.43±0.22

	Grafted bottom（Rootstock）	2	2.37±0.43
	Grafted bottom（Rootstock）	6	1.34±0.09
	Grafted bottom（Rootstock）	12	0.68±0.04
