# Supplementary material for: Plant grafting relieves asymmetry of jasmonic acid response induced by wounding between scion and rootstock in tomato hypocotyl
Source: PLoS One. 2020 Nov 24;15(11):e0241317. doi: 10.1371/journal.pone.0241317 (PMC7685457; doi:10.1371/journal.pone.0241317)
Supplement: S2 Table — (PDF) [file pone.0241317.s002.pdf]

S2 Table

Genes	Groups	Time (h)	Expression±SE
SlTomLoxD	Separated top	0	0.18±0.01
	Separated top	0.5	18.58±1.58
	Separated top	1	5.49±0.51
	Separated top	2	1.77±0.14
	Separated top	6	0.55±0.04
	Separated top	12	0.47±0.05
	Separated bottom	0	1.00±0.06
	Separated bottom	0.5	23.74±1.77
	Separated bottom	1	8.73±1.26
	Separated bottom	2	1.38±0.09
	Separated bottom	6	0.22±0.04
	Separated bottom	12	0.02±0.00
	Grafted top (Scion)	0.5	14.59±0.56
	Grafted top (Scion)	1	16.05±1.10
	Grafted top (Scion)	2	4.46±0.42
	Grafted top (Scion)	6	0.47±0.02
	Grafted top (Scion)	12	0.05±0.00
	Grafted bottom（Rootstock）	0.5	42.52±2.02
	Grafted bottom（Rootstock）	1	25.79±0.70
	Grafted bottom（Rootstock）	2	7.22±1.02
	Grafted bottom（Rootstock）	6	0.47±0.02
	Grafted bottom（Rootstock）	12	0.12±0.01
SlLOX11	Separated top	0	0.35±0.01
	Separated top	0.5	1.57±0.02
	Separated top	1	0.77±0.06
	Separated top	2	1.52±0.07
	Separated top	6	2.14±0.07
	Separated top	12	2.97±0.26
	Separated bottom	0	1.00±0.02
	Separated bottom	0.5	0.98±0.01
	Separated bottom	1	1.37±0.07
	Separated bottom	2	1.57±0.08
	Separated bottom	6	1.61±0.16
	Separated bottom	12	0.12±0.01
	Grafted top (Scion)	0.5	0.78±0.01
	Grafted top (Scion)	1	0.76±0.00
	Grafted top (Scion)	2	1.18±0.09
	Grafted top (Scion)	6	2.17±0.08
	Grafted top (Scion)	12	0.03±0.00
	Grafted bottom（Rootstock）	0.5	1.38±0.05
	Grafted bottom（Rootstock）	1	1.69±0.09

	Grafted bottom（Rootstock）	2	2.20±0.21
	Grafted bottom（Rootstock）	6	3.13±0.12
	Grafted bottom（Rootstock）	12	0.17±0.00
SlAOS	Separated top	0	0.38±0.03
	Separated top	0.5	0.88±0.03
	Separated top	1	0.98±0.03
	Separated top	2	1.10±0.07
	Separated top	6	1.38±0.12
	Separated top	12	0.90±0.05
	Separated bottom	0	1.01±0.10
	Separated bottom	0.5	0.96±0.04
	Separated bottom	1	1.01±0.06
	Separated bottom	2	1.13±0.10
	Separated bottom	6	0.86±0.08
	Separated bottom	12	0.08±0.01
	Grafted top (Scion)	0.5	0.64±0.05
	Grafted top (Scion)	1	1.74±0.08
	Grafted top (Scion)	2	2.51±0.20
	Grafted top (Scion)	6	1.68±0.18
	Grafted top (Scion)	12	0.10±0.00
	Grafted bottom（Rootstock）	0.5	1.28±0.11
	Grafted bottom（Rootstock）	1	2.35±0.01
	Grafted bottom（Rootstock）	2	2.07±0.14
	Grafted bottom（Rootstock）	6	1.31±0.05
	Grafted bottom（Rootstock）	12	0.16±0.01
SlOPCL1	Separated top	0	0.42±0.02
	Separated top	0.5	3.34±0.20
	Separated top	1	3.04±0.16
	Separated top	2	1.02±0.05
	Separated top	6	0.49±0.02
	Separated top	12	0.64±0.03
	Separated bottom	0	1.01±0.09
	Separated bottom	0.5	5.59±0.65
	Separated bottom	1	4.38±1.02
	Separated bottom	2	1.21±0.07
	Separated bottom	6	0.29±0.04
	Separated bottom	12	0.02±0.00
	Grafted top (Scion)	0.5	3.39±0.41
	Grafted top (Scion)	1	5.96±0.22
	Grafted top (Scion)	2	2.17±0.05
	Grafted top (Scion)	6	0.54±0.01
	Grafted top (Scion)	12	0.18±0.00
	Grafted bottom（Rootstock）	0.5	8.75±0.26
	Grafted bottom（Rootstock）	1	14.80±1.43

	Grafted bottom（Rootstock）	2	6.43±0.39
	Grafted bottom（Rootstock）	6	0.40±0.01
	Grafted bottom（Rootstock）	12	0.22±0.00
SlOPR3	Separated top	0	1.03±0.06
	Separated top	0.5	3.12±0.28
	Separated top	1	3.26±0.11
	Separated top	2	1.57±0.15
	Separated top	6	0.75±0.03
	Separated top	12	1.02±0.03
	Separated bottom	0	1.00±0.05
	Separated bottom	0.5	5.27±0.51
	Separated bottom	1	4.47±0.42
	Separated bottom	2	2.34±0.07
	Separated bottom	6	0.28±0.02
	Separated bottom	12	0.05±0.00
	Grafted top (Scion)	0.5	3.70±0.17
	Grafted top (Scion)	1	9.23±0.36
	Grafted top (Scion)	2	4.72±0.37
	Grafted top (Scion)	6	0.91±0.05
	Grafted top (Scion)	12	0.17±0.00
	Grafted bottom（Rootstock）	0.5	8.89±0.56
	Grafted bottom（Rootstock）	1	21.60±1.88
	Grafted bottom（Rootstock）	2	11.31±1.39
	Grafted bottom（Rootstock）	6	1.25±0.11
	Grafted bottom（Rootstock）	12	0.24±0.01
SlAOC	Separated top	0	0.76±0.06
	Separated top	0.5	4.21±0.09
	Separated top	1	7.02±0.53
	Separated top	2	6.67±0.20
	Separated top	6	2.12±0.19
	Separated top	12	1.08±0.07
	Separated bottom	0	1.00±0.06
	Separated bottom	0.5	6.81±0.08
	Separated bottom	1	7.37±0.55
	Separated bottom	2	7.29±0.44
	Separated bottom	6	0.61±0.01
	Separated bottom	12	0.05±0.00
	Grafted top (Scion)	0.5	4.69±0.19
	Grafted top (Scion)	1	16.04±2.05
	Grafted top (Scion)	2	15.67±1.47
	Grafted top (Scion)	6	1.32±0.28
	Grafted top (Scion)	12	0.19±0.01
	Grafted bottom（Rootstock）	0.5	8.90±0.35
	Grafted bottom（Rootstock）	1	25.52±2.48

	Grafted bottom（Rootstock）	2	19.08±1.19
	Grafted bottom（Rootstock）	6	1.51±0.11
	Grafted bottom（Rootstock）	12	0.25±0.00
SlJAZ1	Separated top	0	1.50±0.47
	Separated top	0.5	19.18±2.83
	Separated top	1	17.19±2.15
	Separated top	2	11.23±1.04
	Separated top	6	3.80±1.31
	Separated top	12	3.42±0.40
	Separated bottom	0	1.00±0.03
	Separated bottom	0.5	45.93±1.41
	Separated bottom	1	15.25±4.71
	Separated bottom	2	10.15±2.61
	Separated bottom	6	1.10±0.12
	Separated bottom	12	0.32±0.04
	Grafted top (Scion)	0.5	19.50±1.35
	Grafted top (Scion)	1	34.22±2.30
	Grafted top (Scion)	2	9.59±0.91
	Grafted top (Scion)	6	4.72±0.49
	Grafted top (Scion)	12	1.82±0.23
	Grafted bottom（Rootstock）	0.5	33.64±0.03
	Grafted bottom（Rootstock）	1	40.49±8.29
	Grafted bottom（Rootstock）	2	23.23±2.87
	Grafted bottom（Rootstock）	6	3.53±0.79
	Grafted bottom（Rootstock）	12	2.47±0.38
SlJAZ2	Separated top	0	0.04±0.00
	Separated top	0.5	11.71±0.59
	Separated top	1	7.68±0.53
	Separated top	2	2.84±0.22
	Separated top	6	0.38±0.02
	Separated top	12	0.21±0.02
	Separated bottom	0	1.00±0.07
	Separated bottom	0.5	24.16±3.06
	Separated bottom	1	14.05±1.57
	Separated bottom	2	3.61±0.46
	Separated bottom	6	0.13±0.46
	Separated bottom	12	0.08±0.00
	Grafted top (Scion)	0.5	10.71±0.44
	Grafted top (Scion)	1	18.16±1.19
	Grafted top (Scion)	2	2.75±0.31
	Grafted top (Scion)	6	0.34±0.05
	Grafted top (Scion)	12	0.06±0.00
	Grafted bottom（Rootstock）	0.5	25.36±1.36
	Grafted bottom（Rootstock）	1	24.94±1.98

	Grafted bottom（Rootstock）	2	12.29±0.66
	Grafted bottom（Rootstock）	6	0.77±0.03
	Grafted bottom（Rootstock）	12	0.14±0.01
SlJAZ3	Separated top	0	0.65±0.02
	Separated top	0.5	4.77±0.30
	Separated top	1	4.18±0.24
	Separated top	2	2.54±0.26
	Separated top	6	1.10±0.05
	Separated top	12	0.58±0.07
	Separated bottom	0	1.01±0.11
	Separated bottom	0.5	7.57±0.33
	Separated bottom	1	4.89±0.18
	Separated bottom	2	2.41±0.29
	Separated bottom	6	0.34±0.01
	Separated bottom	12	0.06±0.00
	Grafted top (Scion)	0.5	4.77±0.30
	Grafted top (Scion)	1	4.18±0.24
	Grafted top (Scion)	2	2.54±0.26
	Grafted top (Scion)	6	1.10±0.05
	Grafted top (Scion)	12	0.58±0.07
	Grafted bottom（Rootstock）	0.5	8.80±0.35
	Grafted bottom（Rootstock）	1	20.96±1.66
	Grafted bottom（Rootstock）	2	9.36±0.15
	Grafted bottom（Rootstock）	6	1.30±0.04
	Grafted bottom（Rootstock）	12	0.31±0.03
SlJAZ5	Separated top	0	0.72±0.04
	Separated top	0.5	1.05±0.07
	Separated top	1	2.72±0.10
	Separated top	2	1.11±0.06
	Separated top	6	0.44±0.02
	Separated top	12	0.74±0.01
	Separated bottom	0	1.00±0.07
	Separated bottom	0.5	3.95±0.12
	Separated bottom	1	9.33±0.38
	Separated bottom	2	3.57±0.31
	Separated bottom	6	0.36±0.01
	Separated bottom	12	0.09±0.00
	Grafted top (Scion)	0.5	1.63±0.06
	Grafted top (Scion)	1	4.65±0.25
	Grafted top (Scion)	2	2.23±0.08
	Grafted top (Scion)	6	0.73±0.03
	Grafted top (Scion)	12	0.19±0.02
	Grafted bottom（Rootstock）	0.5	5.52±0.19
	Grafted bottom（Rootstock）	1	15.51±1.06

	Grafted bottom（Rootstock）	2	10.31±0.39
	Grafted bottom（Rootstock）	6	1.03±0.03
	Grafted bottom（Rootstock）	12	0.58±0.00
SlJAZ6	Separated top	0	0.88±0.03
	Separated top	0.5	1.10±0.01
	Separated top	1	2.23±0.20
	Separated top	2	4.78±0.09
	Separated top	6	1.25±0.03
	Separated top	12	0.69±0.01
	Separated bottom	0	1.00±0.06
	Separated bottom	0.5	2.14±0.16
	Separated bottom	1	2.10±0.09
	Separated bottom	2	6.63±0.40
	Separated bottom	6	0.61±0.05
	Separated bottom	12	0.18±0.00
	Grafted top (Scion)	0.5	1.00±0.05
	Grafted top (Scion)	1	2.18±0.06
	Grafted top (Scion)	2	3.19±-0.07
	Grafted top (Scion)	6	1.22±0.02
	Grafted top (Scion)	12	0.26±0.03
	Grafted bottom（Rootstock）	0.5	1.36±0.04
	Grafted bottom（Rootstock）	1	3.13±0.19
	Grafted bottom（Rootstock）	2	3.04±0.12
	Grafted bottom（Rootstock）	6	0.94±0.09
	Grafted bottom（Rootstock）	12	0.45±0.01
SlCOI1	Separated top	0	0.58±0.06
	Separated top	0.5	0.54±0.02
	Separated top	1	0.61±0.09
	Separated top	2	0.86±0.02
	Separated top	6	0.36±0.00
	Separated top	12	0.74±0.05
	Separated bottom	0	1.01±0.10
	Separated bottom	0.5	0.94±0.05
	Separated bottom	1	0.94±0.01
	Separated bottom	2	0.80±0.01
	Separated bottom	6	0.52±0.03
	Separated bottom	12	0.09±0.00
	Grafted top (Scion)	0.5	0.46±0.03
	Grafted top (Scion)	1	0.49±0.01
	Grafted top (Scion)	2	0.55±0.07
	Grafted top (Scion)	6	0.37±0.01
	Grafted top (Scion)	12	0.17±0.01
	Grafted bottom（Rootstock）	0.5	0.61±0.03
	Grafted bottom（Rootstock）	1	0.75±0.01

	Grafted bottom（Rootstock）	2	0.72±0.04
	Grafted bottom（Rootstock）	6	0.50±0.02
	Grafted bottom（Rootstock）	12	0.41±0.01
SlMYC2	Separated top	0	0.24±0.04
	Separated top	0.5	1.78±0.04
	Separated top	1	1.20±0.08
	Separated top	2	0.37±0.05
	Separated top	6	0.46±0.03
	Separated top	12	0.51±0.04
	Separated bottom	0	1.00±0.10
	Separated bottom	0.5	1.28±0.20
	Separated bottom	1	2.33±0.15
	Separated bottom	2	0.48±0.02
	Separated bottom	6	0.53±0.07
	Separated bottom	12	0.03±0.01
	Grafted top (Scion)	0.5	1.50±0.01
	Grafted top (Scion)	1	2.22±0.15
	Grafted top (Scion)	2	0.55±0.05
	Grafted top (Scion)	6	0.54±0.05
	Grafted top (Scion)	12	0.05±0.00
	Grafted bottom（Rootstock）	0.5	4.60±0.21
	Grafted bottom（Rootstock）	1	2.53±0.04
	Grafted bottom（Rootstock）	2	1.37±0.01
	Grafted bottom（Rootstock）	6	0.66±0.03
	Grafted bottom（Rootstock）	12	0.11±0.02
SlPI-1	Separated top	0	0.57±0.02
	Separated top	0.5	0.57±0.01
	Separated top	1	1.62±0.06
	Separated top	2	7.52±0.12
	Separated top	6	11.22±0.28
	Separated top	12	7.00±0.22
	Separated bottom	0	1.00±0.06
	Separated bottom	0.5	1.70±0.16
	Separated bottom	1	0.69±0.02
	Separated bottom	2	0.66±0.01
	Separated bottom	6	0.30±0.02
	Separated bottom	12	0.14±0.02
	Grafted top (Scion)	0.5	0.88±0.01
	Grafted top (Scion)	1	2.71±0.18
	Grafted top (Scion)	2	10.34±0.72
	Grafted top (Scion)	6	13.13±0.36
	Grafted top (Scion)	12	1.09±0.02
	Grafted bottom（Rootstock）	0.5	2.49±0.20
	Grafted bottom（Rootstock）	1	2.78±0.23

	Grafted bottom（Rootstock）	2	3.49±0.19
	Grafted bottom（Rootstock）	6	0.78±0.09
	Grafted bottom（Rootstock）	12	0.67±0.05
SlARG	Separated top	0	0.07±0.00
	Separated top	0.5	0.20±0.01
	Separated top	1	0.31±0.01
	Separated top	2	1.00±0.02
	Separated top	6	5.19±0.01
	Separated top	12	1.19±0.08
	Separated bottom	0	1.00±0.04
	Separated bottom	0.5	0.34±0.06
	Separated bottom	1	0.46±0.04
	Separated bottom	2	1.13±0.13
	Separated bottom	6	0.53±0.04
	Separated bottom	12	0.04±0.01
	Grafted top (Scion)	0.5	0.03±0.00
	Grafted top (Scion)	1	0.14±0.00
	Grafted top (Scion)	2	1.04±0.09
	Grafted top (Scion)	6	3.87±0.32
	Grafted top (Scion)	12	0.10±0.01
	Grafted bottom（Rootstock）	0.5	0.37±0.03
	Grafted bottom（Rootstock）	1	0.46±0.02
	Grafted bottom（Rootstock）	2	0.67±0.04
	Grafted bottom（Rootstock）	6	0.96±0.09
	Grafted bottom（Rootstock）	12	0.32±0.02
SlTD	Separated top	0	0.75±0.11
	Separated top	0.5	1.35±0.06
	Separated top	1	87.71±4.39
	Separated top	2	427.51±20.00
	Separated top	6	558.04±13.91
	Separated top	12	446.65±20.43
	Separated bottom	0	1.01±0.09
	Separated bottom	0.5	10.51±0.70
	Separated bottom	1	147.04±7.05
	Separated bottom	2	239.03±11.32
	Separated bottom	6	39.96±2.31
	Separated bottom	12	1.83±0.38
	Grafted top (Scion)	0.5	1.24±0.05
	Grafted top (Scion)	1	50.57±2.01
	Grafted top (Scion)	2	141.28±9.81
	Grafted top (Scion)	6	280.08±10.27
	Grafted top (Scion)	12	44.21±1.00
	Grafted bottom（Rootstock）	0.5	3.23±0.17
	Grafted bottom（Rootstock）	1	84.13±3.42

	Grafted bottom（Rootstock）	2	365.37±28.20
	Grafted bottom（Rootstock）	6	144.24±5.91
	Grafted bottom（Rootstock）	12	15.42±1.02
SlJA2L	Separated top	0	0.66±0.06
	Separated top	0.5	0.51±0.01
	Separated top	1	2.31±0.09
	Separated top	2	5.48±0.26
	Separated top	6	1.02±0.08
	Separated top	12	5.96±0.14
	Separated bottom	0	1.00±0.01
	Separated bottom	0.5	1.17±0.06
	Separated bottom	1	1.89±0.02
	Separated bottom	2	2.37±0.15
	Separated bottom	6	0.15±0.02
	Separated bottom	12	0.11±0.00
	Grafted top (Scion)	0.5	0.81±0.05
	Grafted top (Scion)	1	2.59±0.16
	Grafted top (Scion)	2	0.93±0.06
	Grafted top (Scion)	6	1.62±0.11
	Grafted top (Scion)	12	0.61±0.02
	Grafted bottom（Rootstock）	0.5	2.18±0.06
	Grafted bottom（Rootstock）	1	5.06±0.13
	Grafted bottom（Rootstock）	2	1.06±0.07
	Grafted bottom（Rootstock）	6	1.37±0.02
	Grafted bottom（Rootstock）	12	0.79±0.07
SlLAPA	Separated top	0	1.83±0.12
	Separated top	0.5	37.07±2.06
	Separated top	1	704.00±13.60
	Separated top	2	2749.63±143.30
	Separated top	6	2859.45±122.14
	Separated top	12	116.22±5.14
	Separated bottom	0	1.00±0.00
	Separated bottom	0.5	72.59±1.46
	Separated bottom	1	396.34±5.73
	Separated bottom	2	366.35±20.81
	Separated bottom	6	7.57±0.28
	Separated bottom	12	0.21±0.00
	Grafted top (Scion)	0.5	19.59±1.63
	Grafted top (Scion)	1	630.92±19.15
	Grafted top (Scion)	2	3822.20±216.32
	Grafted top (Scion)	6	460.25±10.39
	Grafted top (Scion)	12	9.61±1.03
	Grafted bottom（Rootstock）	0.5	35.45±0.31
	Grafted bottom（Rootstock）	1	1254.37±41.97

	Grafted bottom（Rootstock）	2	2795.53±133.02
	Grafted bottom（Rootstock）	6	37.94±1.02
	Grafted bottom（Rootstock）	12	0.65±0.04
SlPR-STH2	Separated top	0	0.66±0.14
	Separated top	0.5	37.97±7.23
	Separated top	1	733.88±42.30
	Separated top	2	130.29±17.61
	Separated top	6	26.18±3.37
	Separated top	12	68.24±5.98
	Separated bottom	0	1.02±0.25
	Separated bottom	0.5	423.58±39.20
	Separated bottom	1	2214.55±131.87
	Separated bottom	2	892.58±34.37
	Separated bottom	6	26.88±2.85
	Separated bottom	12	1.51±0.16
	Grafted top (Scion)	0.5	96.25±7.84
	Grafted top (Scion)	1	2066.35±267.58
	Grafted top (Scion)	2	851.66±34.64
	Grafted top (Scion)	6	33.90±4.22
	Grafted top (Scion)	12	18.22±2.05
	Grafted bottom（Rootstock）	0.5	690.43±48.84
	Grafted bottom（Rootstock）	1	11410.26±2346.49
	Grafted bottom（Rootstock）	2	7321.09±1073.04
	Grafted bottom（Rootstock）	6	35.52±3.56
	Grafted bottom（Rootstock）	12	3.22±0.55
SlERF（Solyc01g090560）	Separated top	0	0.75±0.06
	Separated top	0.5	9.37±0.14
	Separated top	1	5.74±0.24
	Separated top	2	3.07±0.29
	Separated top	6	0.91±0.05
	Separated top	12	1.33±0.14
	Separated bottom	0	1.01±0.08
	Separated bottom	0.5	12.72±0.34
	Separated bottom	1	1.73±0.27
	Separated bottom	2	1.26±0.04
	Separated bottom	6	0.39±0.03
	Separated bottom	12	0.17±0.03
	Grafted top (Scion)	0.5	4.62±0.04
	Grafted top (Scion)	1	13.36±1.20
	Grafted top (Scion)	2	2.84±0.05
	Grafted top (Scion)	6	0.91±0.11
	Grafted top (Scion)	12	0.31±0.01
	Grafted bottom（Rootstock）	0.5	9.61±0.20
	Grafted bottom（Rootstock）	1	22.42±1.76

	Grafted bottom（Rootstock）	2	4.53±0.35
	Grafted bottom（Rootstock）	6	0.43±0.00
	Grafted bottom（Rootstock）	12	0.76±0.09
SlERF（Solyc06g075510）	Separated top	0	0.16±0.00
	Separated top	0.5	1.38±0.03
	Separated top	1	1.17±0.04
	Separated top	2	0.95±0.06
	Separated top	6	0.65±0.03
	Separated top	12	0.36±0.01
	Separated bottom	0	1.00±0.07
	Separated bottom	0.5	4.97±0.12
	Separated bottom	1	1.90±0.13
	Separated bottom	2	2.57±0.07
	Separated bottom	6	0.98±0.05
	Separated bottom	12	0.15±0.02
	Grafted top (Scion)	0.5	0.94±0.05
	Grafted top (Scion)	1	2.52±0.07
	Grafted top (Scion)	2	1.70±0.01
	Grafted top (Scion)	6	0.67±0.01
	Grafted top (Scion)	12	0.36±0.02
	Grafted bottom（Rootstock）	0.5	3.49±0.12
	Grafted bottom（Rootstock）	1	7.36±0.13
	Grafted bottom（Rootstock）	2	4.34±0.15
	Grafted bottom（Rootstock）	6	1.64±0.03
	Grafted bottom（Rootstock）	12	0.51±0.01
SlERF（Solyc02g070040）	Separated top	0	0.58±0.01
	Separated top	0.5	4.21±0.09
	Separated top	1	2.63±0.06
	Separated top	2	2.64±0.14
	Separated top	6	0.59±0.02
	Separated top	12	0.87±0.04
	Separated bottom	0	1.01±0.07
	Separated bottom	0.5	7.13±0.15
	Separated bottom	1	4.57±0.22
	Separated bottom	2	1.58±0.07
	Separated bottom	6	0.36±0.01
	Separated bottom	12	0.27±0.04
	Grafted top (Scion)	0.5	2.88±0.07
	Grafted top (Scion)	1	4.10±0.10
	Grafted top (Scion)	2	0.75±0.04
	Grafted top (Scion)	6	0.81±0.04
	Grafted top (Scion)	12	0.19±0.02
	Grafted bottom（Rootstock）	0.5	3.87±0.02
	Grafted bottom（Rootstock）	1	10.46±0.34

	Grafted bottom（Rootstock）	2	2.24±0.09
	Grafted bottom（Rootstock）	6	0.47±0.05
	Grafted bottom（Rootstock）	12	0.22±0.01
SlERF（Solyc01g090320）	Separated top	0	1.03±0.02
	Separated top	0.5	51.99±4.08
	Separated top	1	90.47±2.48
	Separated top	2	22.06±0.68
	Separated top	6	0.14±0.02
	Separated top	12	0.19±0.02
	Separated bottom	0	1.00±0.06
	Separated bottom	0.5	81.27±5.55
	Separated bottom	1	63.60±2.32
	Separated bottom	2	7.44±0.38
	Separated bottom	6	0.48±0.06
	Separated bottom	12	0.19±0.03
	Grafted top (Scion)	0.5	50.58±1.41
	Grafted top (Scion)	1	30.77±2.72
	Grafted top (Scion)	2	22.06±0.68
	Grafted top (Scion)	6	0.14±0.02
	Grafted top (Scion)	12	0.19±0.02
	Grafted bottom（Rootstock）	0.5	61.68±4.13
	Grafted bottom（Rootstock）	1	233.69±5.17
	Grafted bottom（Rootstock）	2	56.20±2.84
	Grafted bottom（Rootstock）	6	0.42±0.02
	Grafted bottom（Rootstock）	12	2.06±0.35
SlbHLH（Solyc05g050560）	Separated top	0	0.62±0.00
	Separated top	0.5	2.46±0.04
	Separated top	1	1.99±0.03
	Separated top	2	1.03±0.00
	Separated top	6	0.26±0.01
	Separated top	12	0.59±0.02
	Separated bottom	0	1.01±0.10
	Separated bottom	0.5	3.27±0.10
	Separated bottom	1	2.05±0.07
	Separated bottom	2	1.07±0.01
	Separated bottom	6	0.31±0.00
	Separated bottom	12	0.07±0.00
	Grafted top (Scion)	0.5	1.91±0.00
	Grafted top (Scion)	1	5.23±0.14
	Grafted top (Scion)	2	0.98±0.05
	Grafted top (Scion)	6	0.42±0.02
	Grafted top (Scion)	12	0.16±0.00
	Grafted bottom（Rootstock）	0.5	3.28±0.10
	Grafted bottom（Rootstock）	1	7.68±0.18

	Grafted bottom（Rootstock）	2	3.27±0.10
	Grafted bottom（Rootstock）	6	0.45±0.00
	Grafted bottom（Rootstock）	12	0.31±0.02
SlbHLH（Solyc09g083360）	Separated top	0	0.35±0.02
	Separated top	0.5	2.90±0.06
	Separated top	1	1.13±0.09
	Separated top	2	0.40±0.01
	Separated top	6	0.43±0.01
	Separated top	12	1.34±0.04
	Separated bottom	0	1.00±0.00
	Separated bottom	0.5	9.73±0.20
	Separated bottom	1	1.56±0.04
	Separated bottom	2	1.14±0.03
	Separated bottom	6	0.12±0.00
	Separated bottom	12	0.11±0.00
	Grafted top (Scion)	0.5	3.25±0.13
	Grafted top (Scion)	1	1.79±0.02
	Grafted top (Scion)	2	0.47±0.01
	Grafted top (Scion)	6	0.13±0.01
	Grafted top (Scion)	12	0.86±0.04
	Grafted bottom（Rootstock）	0.5	5.93±0.06
	Grafted bottom（Rootstock）	1	5.43±0.11
	Grafted bottom（Rootstock）	2	0.72±0.02
	Grafted bottom（Rootstock）	6	0.26±0.01
	Grafted bottom（Rootstock）	12	0.15±0.00
SlTCP5	Separated top	0	1.12±0.10
	Separated top	0.5	2.30±0.11
	Separated top	1	2.51±0.09
	Separated top	2	3.54±0.07
	Separated top	6	3.33±0.14
	Separated top	12	3.87±0.06
	Separated bottom	0	1.01±0.07
	Separated bottom	0.5	1.55±0.06
	Separated bottom	1	2.31±0.06
	Separated bottom	2	1.12±0.02
	Separated bottom	6	1.79±0.10
	Separated bottom	12	0.32±0.01
	Grafted top (Scion)	0.5	1.54±0.03
	Grafted top (Scion)	1	3.89±0.21
	Grafted top (Scion)	2	4.01±0.10
	Grafted top (Scion)	6	4.38±0.51
	Grafted top (Scion)	12	0.74±0.02
	Grafted bottom（Rootstock）	0.5	1.85±0.02
	Grafted bottom（Rootstock）	1	4.83±0.10

	Grafted bottom（Rootstock）	2	1.91±0.06
	Grafted bottom（Rootstock）	6	4.66±0.08
	Grafted bottom（Rootstock）	12	1.35±0.05
SlHD-Zip	Separated top	0	0.90±0.01
	Separated top	0.5	1.57±0.04
	Separated top	1	0.63±0.05
	Separated top	2	0.70±0.03
	Separated top	6	0.38±0.03
	Separated top	12	0.64±0.00
	Separated bottom	0	1.01±0.07
	Separated bottom	0.5	1.98±0.06
	Separated bottom	1	0.88±0.07
	Separated bottom	2	0.94±0.00
	Separated bottom	6	0.22±0.00
	Separated bottom	12	0.02±0.00
	Grafted top (Scion)	0.5	1.50±0.05
	Grafted top (Scion)	1	1.86±0.04
	Grafted top (Scion)	2	0.79±0.01
	Grafted top (Scion)	6	0.61±0.02
	Grafted top (Scion)	12	0.16±0.00
	Grafted bottom（Rootstock）	0.5	2.83±0.07
	Grafted bottom（Rootstock）	1	4.27±0.13
	Grafted bottom（Rootstock）	2	2.29±0.02
	Grafted bottom（Rootstock）	6	0.39±0.01
	Grafted bottom（Rootstock）	12	0.18±0.01
SlMPK1	Separated top	0	0.45±0.00
	Separated top	0.5	0.52±0.01
	Separated top	1	0.28±0.01
	Separated top	2	0.37±0.03
	Separated top	6	0.44±0.01
	Separated top	12	0.94±0.04
	Separated bottom	0	1.00±0.04
	Separated bottom	0.5	0.59±0.02
	Separated bottom	1	0.67±0.01
	Separated bottom	2	0.29±0.01
	Separated bottom	6	0.46±0.02
	Separated bottom	12	0.01±0.00
	Grafted top (Scion)	0.5	0.47±0.01
	Grafted top (Scion)	1	0.52±0.01
	Grafted top (Scion)	2	0.54±0.01
	Grafted top (Scion)	6	0.77±0.04
	Grafted top (Scion)	12	0.11±0.61
	Grafted bottom（Rootstock）	0.5	1.04±0.06
	Grafted bottom（Rootstock）	1	1.05±0.06

	Grafted bottom（Rootstock）	2	1.18±0.01
	Grafted bottom（Rootstock）	6	0.89±0.02
	Grafted bottom（Rootstock）	12	0.25±0.00
SlMPK2	Separated top	0	0.65±0.02
	Separated top	0.5	0.60±0.02
	Separated top	1	0.38±0.01
	Separated top	2	0.62±0.01
	Separated top	6	0.46±0.03
	Separated top	12	0.69±0.01
	Separated bottom	0	1.00±0.02
	Separated bottom	0.5	1.10±0.09
	Separated bottom	1	0.60±0.01
	Separated bottom	2	0.95±0.01
	Separated bottom	6	0.44±0.01
	Separated bottom	12	0.05±0.00
	Grafted top (Scion)	0.5	0.61±0.02
	Grafted top (Scion)	1	0.65±0.03
	Grafted top (Scion)	2	0.57±0.03
	Grafted top (Scion)	6	0.60±0.01
	Grafted top (Scion)	12	0.42±0.01
	Grafted bottom（Rootstock）	0.5	0.95±0.05
	Grafted bottom（Rootstock）	1	1.34±0.03
	Grafted bottom（Rootstock）	2	1.19±0.01
	Grafted bottom（Rootstock）	6	0.78±0.03
	Grafted bottom（Rootstock）	12	0.44±0.01
SlMPK3	Separated top	0	0.39±0.01
	Separated top	0.5	4.56±0.22
	Separated top	1	0.43±0.02
	Separated top	2	0.12±0.01
	Separated top	6	0.05±0.00
	Separated top	12	0.15±0.00
	Separated bottom	0	1.01±0.10
	Separated bottom	0.5	6.96±0.18
	Separated bottom	1	0.51±0.01
	Separated bottom	2	0.23±0.01
	Separated bottom	6	0.10±0.00
	Separated bottom	12	0.01±0.00
	Grafted top (Scion)	0.5	2.99±0.10
	Grafted top (Scion)	1	0.56±0.02
	Grafted top (Scion)	2	0.16±0.00
	Grafted top (Scion)	6	0.10±0.01
	Grafted top (Scion)	12	0.03±0.00
	Grafted bottom（Rootstock）	0.5	8.55±0.16
	Grafted bottom（Rootstock）	1	1.46±0.02

	Grafted bottom（Rootstock）	2	0.59±0.00
	Grafted bottom（Rootstock）	6	0.12±0.01
	Grafted bottom（Rootstock）	12	0.04±0.00
