# Supplementary material for: Plant grafting relieves asymmetry of jasmonic acid response induced by wounding between scion and rootstock in tomato hypocotyl
Source: PLoS One. 2020 Nov 24;15(11):e0241317. doi: 10.1371/journal.pone.0241317 (PMC7685457; doi:10.1371/journal.pone.0241317)
Supplement: S3 Table — (PDF) [file pone.0241317.s003.pdf]

S3 Table

Gene Symbol	GeneBank	Forward primer sequence (5'→3')	Reverse primer sequence (5'→3')
SlTUA	XM_010320721.2	ATGAGATTTGCCATCAGGG	ATTGCATGACAAGGACCG
SlERF	Solyc01g090320	ACACGCCAGAAGATTGGA	GCCTCGCACCTTTCTTATT
SlERF	Solyc01g090560	TGTACTCTTGAGAAGAGACCTG	GAGAAATACACACACGATGGA
SlERF	Solyc02g070040	GAGATCCTCTGGAGTCGAAAT	ACTTGACTCTTCTTGCTGTAAT
SlTCP 5	Solyc02g089020	GGCTAAGAGATCGTCGTGT	GGTTGATTAAGCCCTAATCTGT
SlERF	Solyc06g075510	TGACAATGTTCTGCACGG	AGGCATTTGTTGAAGTTGCTA
SlHD-Zip	Solyc05g051460	GTTCTTGGAATCGAATGAGGT	ACTGCAAGATTCTTCGCC
SlbHLH	Solyc09g083360	GAGTGGAGCAGCAGAGAT	CTTTCTCAATGGGCACGA
SlbHLH	Solyc05g050560	GAAACAGGGCTCCCATCATA	GTTCCCTGGCTTTACATTACC
SlJAZ2	Solyc12g009220	CAATTGCACCATACCAAATCT	TTGAGCACCTAATCCCAACC
SlTomLoxD	Solyc03g122340	TTGGAAATCCAGATAGAGGCAT	TGTGTCTGTAGGAACACGG
SlOPR3	Solyc07g007870	TCAAACAACAATGGCGTCT	CATCGGAGCCAATACTACC
SlJAZ5	Solyc03g118540	TGTGTATGACAATGTTTCACCG	TATGGCCGAGGACTTAGGTA
SlAOC	Solyc02g085730	CAGAGCACCTCAACAGATTC	CTTCCACGATCACGTTCATT
SlAOS	Solyc11g069800	GGAAACTAAGCTCGGAACTG	CGGAAGACCGAGAGTTATC
SlPR-STH2	Solyc05g054380	TTCCCCTATAACACCAATTCGT	ACTTGTTCAATACTTCCAGCTC
SlPI-1	Solyc09g084470	ATTGATGGGCCAGAAGTCATA	TAGCAAGCTTTGTTGGTACAC
SlJAZ6	Solyc01g005440	GCGACTACCATAATGACAGC	GTTCATCACCCTTTCCTTGC
SlJAZ3	Solyc03g122190	TGATGATTTCCCTGCTGACAA	TAGCCTGGAAAGTCCCAAATG
SlTD	Solyc09g008670	GCAAGTGGAGCTAATATGGAT	AGCTTCCTTGTTGTTCTACC

SlJA2L	Solyc07g063410	CTCGACGATTGGGTTCTATG	CCTTGTTCTGTAAATCAGAGCA
SlJAZ1	Solyc07g042170	GCGAGAAGAGCTTCATTAACA	TGCTGCTTCCTCTCTATGG
SlLAPA	Solyc12g010020	GGAAAGTCTGGACAATCCGT	GCCTGTCCTAAACTGTGATAAG
SlARG	Solyc01g091170	AATTGCTAACGTCAAATGTTCC	ACGAATAAGTGTAAGAGATGCC
SlMYC2	Solyc08g076930	GTCGGTTGTTGATTTCTCAAGT	AGCAATATAAGCAGGTGATGAC
SlLOX11	Solyc05g014790	ACGATTTAGGCAATCCTGAC	CTTTCTTAGTTGGACCTCTACC
SlOPCL1	Solyc12g094520	ATTTGTCTCGCCGTTATGTCA	TCTGTTTAGCGATTTCGCTG
SlMPK1	Solyc12g019460	CACAGAGAGAAGCCTTTAACG	ATAAACCCTGATTCGAGCG
SlMPK2	Solyc08g014420	ACCAGCTACGTCTGCTTA	ATACCGCTTTGCATTCTCAT
SlMPK3	Solyc06g005170	AACTGAGCTTCTTGGCAC	AATGGATTCACATGAGGGAAC
SlCOI1	Solyc05g052620	GGATGCTTCTGGGATACG	CAAGGTAGCCAAGGCTAATG
