# Supplementary figures and images for: Plant grafting relieves asymmetry of jasmonic acid response induced by wounding between scion and rootstock in tomato hypocotyl
Source: PLoS One. 2020 Nov 24;15(11):e0241317. doi: 10.1371/journal.pone.0241317 (PMC7685457; doi:10.1371/journal.pone.0241317)

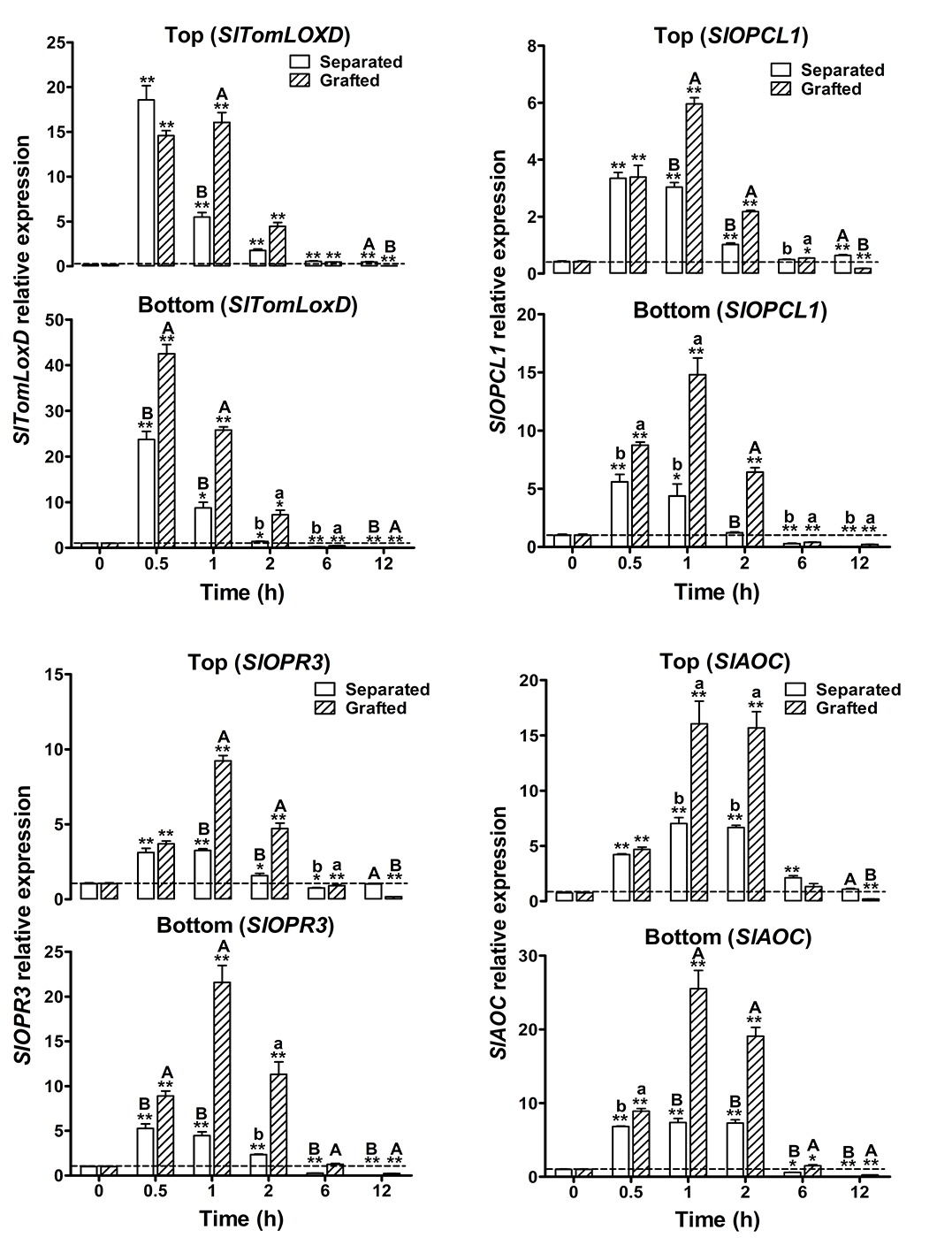

Supplement: S1 Fig — (TIF) [file pone.0241317.s004.tif]

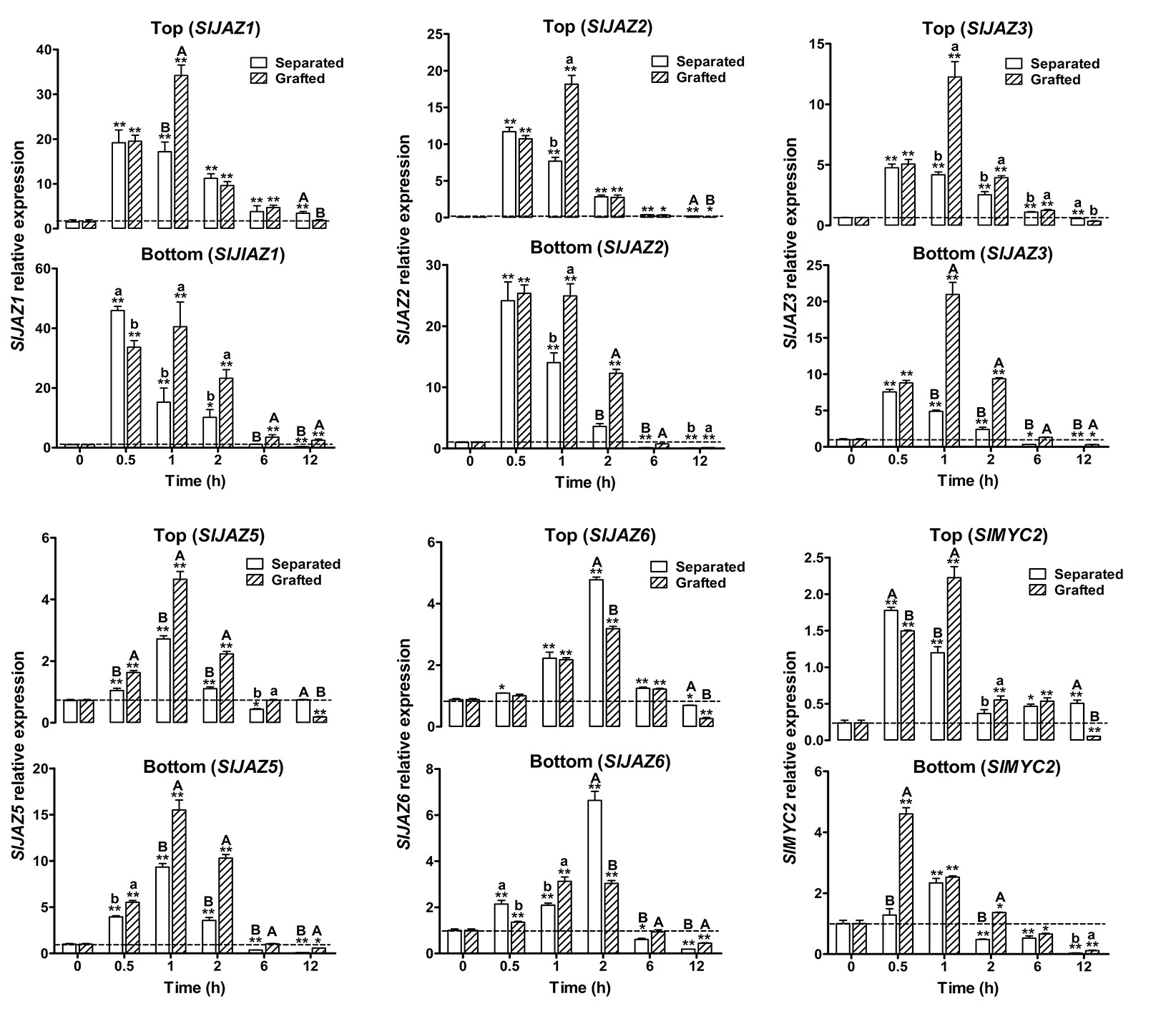

Supplement: S2 Fig — (TIF) [file pone.0241317.s005.tif]

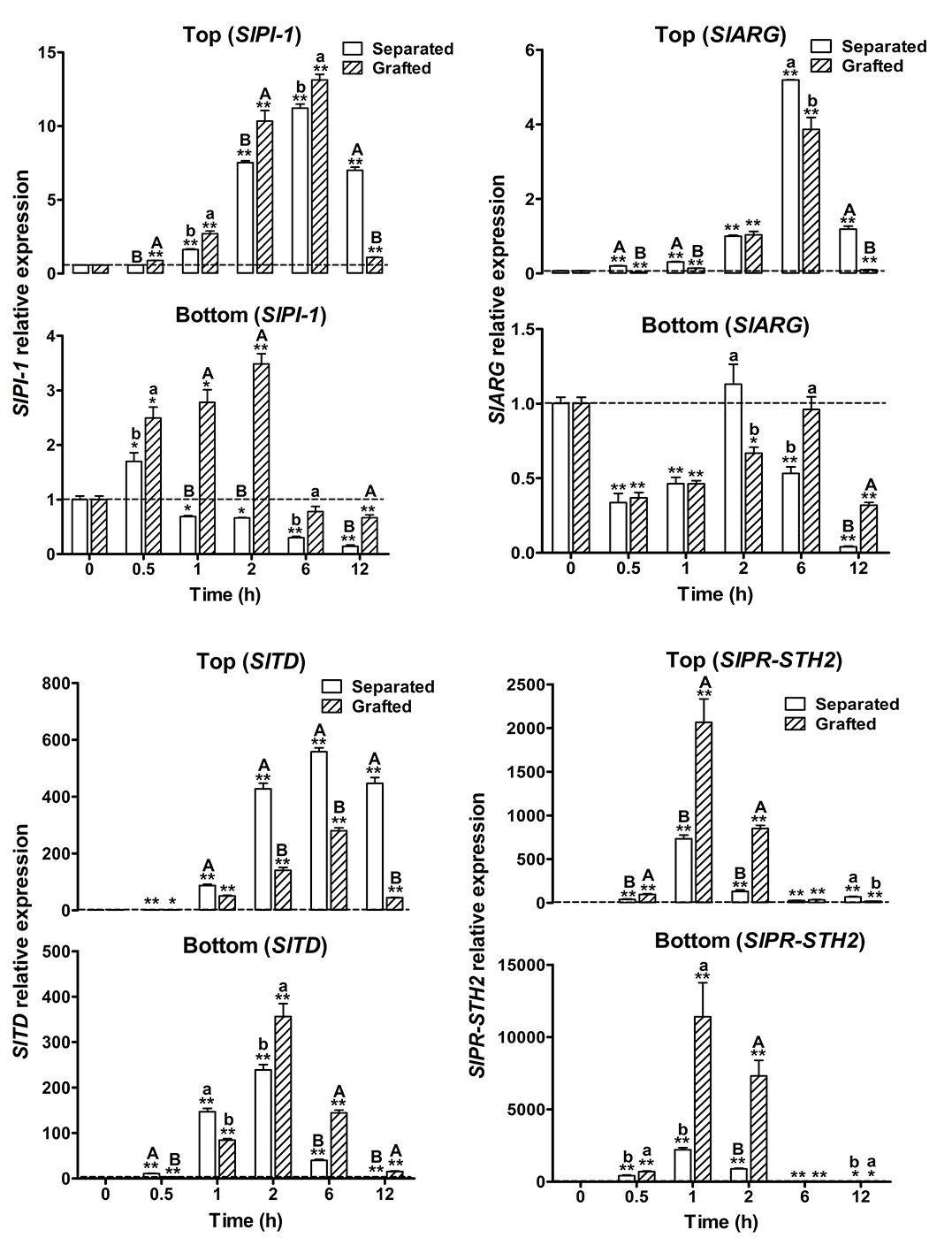

Supplement: S3 Fig — (TIF) [file pone.0241317.s006.tif]

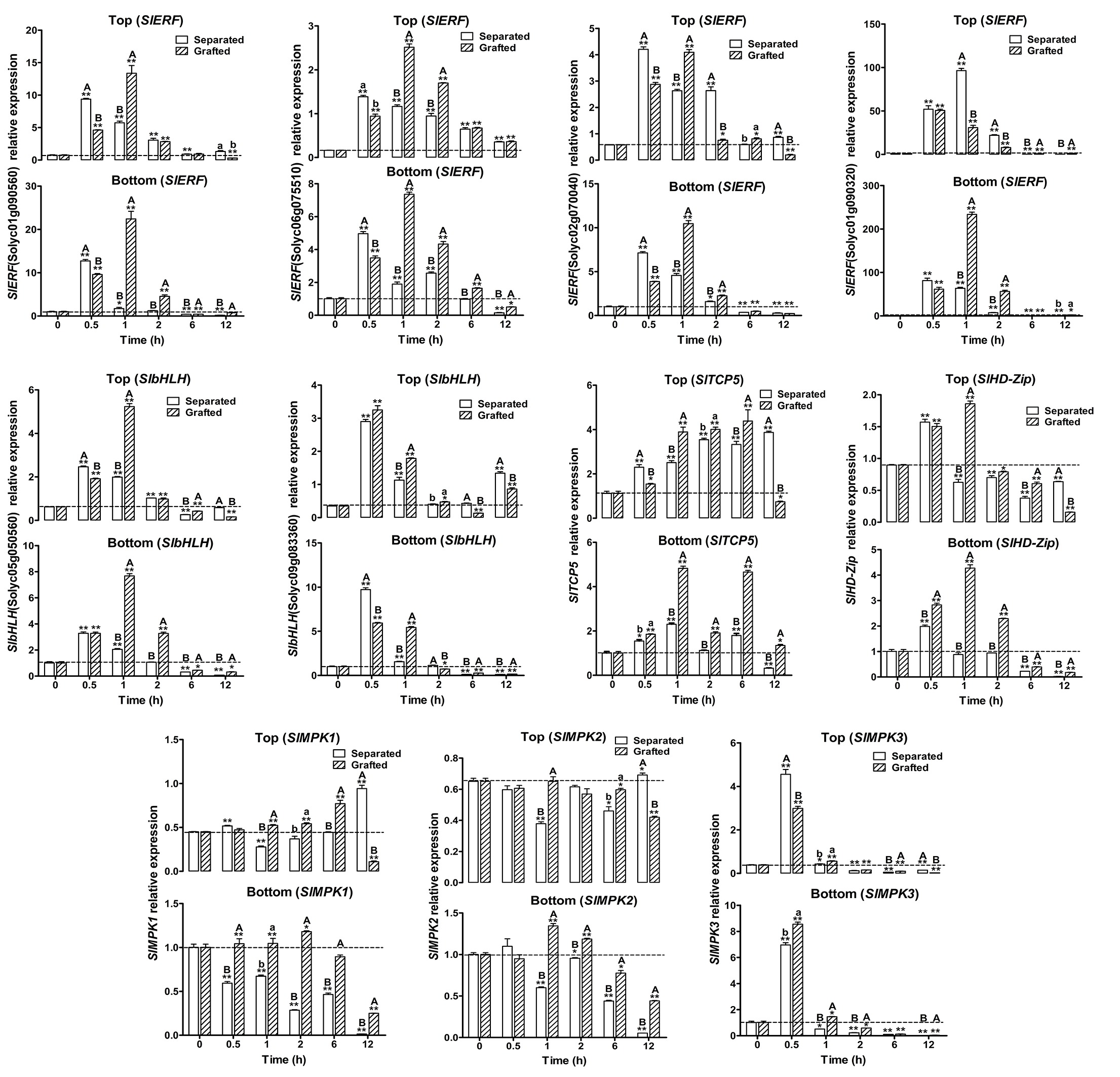

Supplement: S4 Fig — (TIF) [file pone.0241317.s007.tif]
